# Percutaneous edge‐to‐edge repair of severe mitral regurgitation using the MitraClip XTR versus NTR system

**DOI:** 10.1002/clc.23599

**Published:** 2021-03-24

**Authors:** Philipp M. Doldi, Isabel Brinkmann, Mathias Orban, Lukas Stolz, Martin Orban, Thomas Stocker, Kornelia Loew, Joscha Buech, Michael Nabauer, Ben Illigens, Tiago Lemos Cerqueira, Timo Siepmann, Steffen Massberg, Joerg Hausleiter, Daniel Braun

**Affiliations:** ^1^ Medizinische Klinik und Poliklinik I University Hospital Munich Campus Grosshadern Marchioninistraße München Deutschland Germany; ^2^ German Sites Development Principles and Practice of Clinical Research Harvard T.H. Chan School of Public Health Dresden International University Dresden Germany; ^3^ Herzchirurgische Klinik und Poliklinik University Hospital Munich Campus Grosshadern Marchioninistraße München Deutschland Germany; ^4^ Department of Neurology University Hospital Carl Gustav Carus at the Technische Universität Dresden Dresden Germany; ^5^ Division of Health Care Sciences Center for Clinical Research and Management Education Dresden Dresden International University Dresden Germany

**Keywords:** leaflet injury, mitral valve regurgitation, single leaflet device attachment, transcatheter mitral valve repair

## Abstract

**Background:**

Transcatheter mitral valve repair (TMVR) has shown to improve symptoms and functional capacity in patients with severe mitral valve regurgitation (MR). Novel device developments provide the technology to treat patients with complex anatomies and large coaptation gaps. Nevertheless, the question of superiority of one device remains unanswered. We aimed to compare the MitraClip XTR and MitraClip NTR system in a real world setting.

**Hypothesis:**

TMVR with the MitraClip XTR system is equally effective, but associated with a higher risk of leaflet injury.

**Methods:**

We retrospectively analyzed peri‐procedural and mid‐term clinical and echocardiographic outcomes of 113 patients treated for severe MR between March 2018 and August 2019 at the University Hospital of Munich.

**Results:**

Postprocedural MR reduction to ≤2+ was comparable in both groups (XTR: 96.1% vs. NTR: 97.6%, *p =* .38). There was a significant difference in a composite safety endpoint of periprocedural Major adverse cardiac and cerebrovascular events (MACCE) including leaflet injury between groups (XTR 14.6% vs. NTR 1.7%, 95% CI [2.7, 24.6], *p =* .012). After a median follow‐up of 8.5 (4.4, 14.0) months, durable reduction of MR was confirmed (XTR: in 91.9% vs. NTR: 96.8%, *p =* .31) and clinical and symptomatic improvement was comparable in both groups accordingly.

**Conclusion:**

While efficacy was comparable in both treatment groups, patients treated with the MitraClip XTR systems showed more events of acute leaflet tear and single leaflet device attachment (SLDA). A detailed echocardiographic assessment should be done to identify risk candidates for acute leaflet injury.

## 1 INTRODUCTION

Heart failure (HF) and related symptoms often originate from severe MR. Being the second most frequent heart valve disease in our society, MR represents an important health challenge.^1^, ^2^ It is acknowledged that two different entities of MR should be distinguished: Primary MR (degenerative) and secondary (functional) MR.^3^ Given these different etiologies, a precise characterization of the disease is mandatory to decide on an adequate treatment approach.^4^

Surgical mitral valve repair remains the gold‐standard treatment for patients with primary MR and acceptable operative risk.^5^, ^6^, ^7^ For patients with a highly elevated perioperative risk of mortality the MitraClip system (Abbott Vascular, Abbott Park, Illinois) for transcatheter mitral valve repair (TMVR) has emerged as an alternative treatment approach.^8^, ^9^ Surgical results frequently show suboptimal results with recurrence of MR after surgical mitral valve repair for secondary MR.^10^ Therefore, a large proportion of patients with severe secondary MR are currently treated by edge‐to‐edge‐repair. Patients with secondary MR seem to benefit from TMVR in terms of mortality and reduction of HF hospitalizations.^8^, ^11^, ^12^

Despite the fact that TMVR showed an effective and durable reduction of MR severity with concomitant symptomatic improvement, the presence of complex anatomic settings including patients with large flails and significant coaptation gaps or patients with significant annulus dilatation and restrictive leaflets complicate leaflet grasping.

The introduction of the new generation MitraClip XTR system with 3 mm longer clip arms enables the treatment of many of these anatomically challenging cases.^13^ Moreover, the availability of the MitraClip XTR system might improve the annuloplasty effect of edge‐to‐edge treatment and might reduce the need of multiple clip interventions by grasping a larger proportion of the mitral leaflets.

However, some authors hypothesized that these adjustments result in increased tension on the leaflets and therefore elevated risk of acute leaflet injury.^13^, ^14^, ^15^ Therefore, the present study aims to compare the efficacy and safety of both MitraClip devices in a real‐world setting.

## 2 METHODS

### 2.1 Study design

We retrospectively analyzed a cohort of 113 consecutive patients with severe symptomatic MR that underwent TMVR at the University Hospital of Munich between March 2018 and August 2019. Out of these, 55 were treated with the MitraClip XTR system and 58 patients were treated with the MitraClip NTR system (Abbott Vascular). Patients treated with a combination of both devices, MitraClip XTR and MitraClip NTR, were considered to be in the XTR group. The objective of this study was the comparison of TMVR using the MitraClip NTR system and the MitraClip XTR system in terms of efficacy and safety. Primary outcome was defined as acute procedural success (MR ≤2+). As a composite safety endpoint, we additionally assessed periprocedural major adverse cardiac and cerebrovascular events (MACCE: Death from any cause, cardiac death, cerebrovascular event, myocardial infarction and leaflet injury (leaflet tear and single leaflet device attachment [SLDA]). Additionally, we analyzed mid‐term survival of both groups.

Secondary outcome measures were defined as MR severity as well as echocardiographic parameters of right and left ventricular function at last available follow‐up. In addition, we assessed functional performance including New York Heart Association (NYHA) functional class and 6‐min walking distance, as well as NT‐proBNP as a laboratory parameter of HF. Follow‐up visits were scheduled for 30 days, 6 months, and 12 months after TMVR.

### 2.2 Study cohort

Patients undergoing TMVR for severe MR were consecutively included into the EVERY‐Valve registry between March 2018 and August 2019 at the University Hospital of Munich that was approved by the local ethics committee. The study was conducted according to international rules for scientific studies as well as the declaration of Helsinki.

All patients showed heart‐failure related symptoms (NYHA II‐IV) despite optimal medical therapy. An interdisciplinary heart team consensus was obtained before TMVR in order to evaluate the best treatment option for each individual.

### 2.3 Echocardiography and procedural techniques

All echocardiographies were performed and analyzed by experienced physicians. Baseline and follow‐up MR severity was assessed according to current recommendations of the American Society of Echocardiography.^16^ Right ventricular parameters were assessed in an apical four‐chamber view.^17^, ^18^, ^19^ The TMVR procedures were performed under general anesthesia with two‐ and three‐dimensional transesophageal echocardiography as well as fluoroscopic guidance as previously described.^20^ The decision of using either the MitraClip XTR or the MitraClip NTR system was up to the preference of the interventional cardiologist.

All patients with symptomatic severe mitral valve regurgitation eligible for TMVR have been included in the study. Additionally, we included patients with planned concomitant interventional tricuspid valve repair. Patients were excluded from the study if other devices than the MitraClip NTR or MitraClip XTR system have been implanted. In case of concomitant transcatheter tricuspid valve repair, complications associated with the tricuspid valve were not registered. After TMVR, routine clinical follow‐up was assessed at 30 days, 3, 6, and 12 months. Last available follow‐up was used to analyze mid‐term follow‐up. Survival information was collected via medical records, telephone calls and the local residents' registration office.

### 2.4 Statistical analysis

For the purpose of descriptive statistics, all numerical continuous data are presented as means or medians with SD and interquartile ranges (IQR) or odds ratios with 95% confidence intervals (CI) respectively, as measures of dispersion. Categorical data are presented in the form of proportions, frequencies or percentages. Normality of data distribution was assessed graphically and using the Shapiro–Wilk test. Comparisons between groups were performed using the Chi‐squared‐test for categorical variables, and Student's *t*‐test or Mann–Whitney‐U test for unpaired continuous variables, and Wilcoxon rank sum test for paired variables, according to data distribution.

*p*‐values are reported with three decimal points; all our tests yield 2‐sided *p*‐values with a level of significance (alpha) of <.05 to determine statistical significance. The statistical software applied for data analysis and visualization was an up‐to‐date version of R (The R Foundation for Statistical Computing, Vienna, Austria). Investigations were in accordance with the Declaration of Helsinki.

## 3 RESULTS

A total number of 113 consecutive patients with a median age of 78 [72, 82] years were considered to have an elevated perioperative mortality with a median EuroScore II of 4.1 [2.8, 7.8] and an STS risk score of 3.5 [2.3, 5.9]. All patients were symptomatic and had MR grade ≥ 3+. Forty‐seven percent of patients suffered from severe secondary MR while 40% of the patients showed a primary etiology of MR. While most characteristics were equally distributed, patients in the NTR group showed a higher occurrence of atrial fibrillation/flutter (XTR: 62.0% vs. NTR: 83%, *p =* .02). Moreover, patients in the XTR group showed higher baseline values for the 6‐min walk distance (231 vs. 188 min, *p =* .02, Table 1).

**TABLE 1:** Clinical characteristics of the study cohort

Characteristic	Overall	XTR	NTR	*p* value
*n*	113	55	58	
Age, years (median [IQR])	78.00 [72.00, 82.00]	78.00 [71.50, 82.00]	77.50 [73.00, 82.00]	.80
Gender, male (%)	73 (64.6)	41 (74.5)	32 (55.2)	.05
EuroSCORE II (median [IQR])	4.13 [2.77, 7.76]	3.88 [2.33, 8.07]	4.35 [3.18, 7.40]	.45
STS score (median [IQR])	3.48 [2.25, 5.89]	3.20 [1.93, 5.59]	3.83 [2.54, 5.92]	.25
MLHFQ score (mean [SD])	38.44 (15.59)	36.53 (18.19)	39.98 (13.12)	.31
6 min walk‐test (median [IQR])	200.0 [131.0, 292.50]	231.0 [163.0, 375.0]	187.5 [103.5, 234.5]	.02
MR etiology				
Functional (%)	50 (47.2)	20 (41.7)	30 (51.7)	.40
Degenerative (%)	42 (39.6)	21 (43.8)	21 (36.2)	.56
Mixed (%)	13 (12.3)	7 (14.6)	6 (10.3)	.72
Mitral valve regurgitation grade (%)				
II°	5 (4.7)	2 (4.2)	3 (5.2)	1.00
III°	61 (57.5)	26 (54.2)	35 (60.3)	.66
IV°	39 (36.8)	20 (41.7)	19 (32.8)	.46
Presence of TR ≥ II (%)	48 (55.2)	24 (47.1)	24 (66.7)	.11
Concominat treatment for TR (%)	28 (26.7)	10 (20.8)	18 (31.6)	.31
NYHA functional class (%)				
II°	7 (6.2)	6 (10.9)	1 (1.7)	.10
III°	78 (69.0)	34 (61.8)	44 (75.9)	.16
IV°	27 (23.9)	15 (27.3)	12 (20.7)	.55
NTproBNP, pg/ml (median [IQR])	3447.0 [1679, 6305]	3447.0 [1568, 7175]	3429.0 [1931, 5942]	.84
previous myocardial infarction (%)	35 (31.2)	17 (30.9)	18 (31.6)	1.00
Previous CABG (%)	11 (10.5)	4 (8.5)	7 (12.1)	.79
Previous PCI (%)	92 (81.4)	44 (80.0)	48 (82.8)	.89
History of atrial fibrillation/flutter (%)	82 (72.6)	34 (61.8)	48 (82.8)	.02
Previous CRT (%)	15 (13.3)	7 (12.7)	8 (13.8)	1.00
ACE Inhibitors (%)	40 (37.7)	19 (39.6)	21 (36.2)	.88
Angiotensine‐receptor blockers (%)	35 (33.0)	17 (35.4)	18 (31.0)	.79
Betablockers (%)	96 (85.7)	49 (90.7)	47 (81.0)	.23
Loop diuretics (%)	89 (85.6)	40 (87.0)	49 (84.5)	.94
Aldosteron antagonists (%)	42 (37.2)	14 (25.5)	28 (48.3)	.02
Thiacide diuretics (%)	16 (15.4)	7 (15.2)	9 (15.5)	1.00
Anticoagulant therapy (%)	93 (87.7)	41 (85.4)	52 (89.7)	.72
COPD (%)	31 (27.4)	17 (30.9)	14 (24.1)	.55
Renal impairment (%)	36 (31.9)	13 (23.6)	23 (39.7)	.10
Presence of EVEREST II inclusion criteria	62 (62.6)	27 (60.0)	35 (64.8)	.78

Concerning echocardiographic baseline measurements, patients in the XTR group showed a higher mitral regurgitant volume (Reg. Vol., 59.4 ml ± 34.2 vs. 44.2 ml ± 28.5, *p =* .02) and a higher effective regurgitant orifice area (EROA, 0.42 cm^2^ ± 0.25 vs. 0.31 cm^2^ ± 0.19, *p =* .02). Accordingly, mitral annular dimensions were significantly larger in the XTR group (36.7 mm ± 4.7 vs. 33.4 mm ± 4.0, *p <* .001). Conversely, patients treated with the MitraClip NTR device initially showed more cases of concomitant high grade TR (TR ≥3+: 47% vs. 22%, *p =* .04). All echocardiographic baseline parameters are given in Table 2.

**TABLE 2:** Echocardiographic baseline parameters of the study cohort

Characteristic	Overall	XTR	NTR	*p* value
*n*	113	55	58	
LVEF (%) (mean (SD))	47.6 (15.6)	48.2 (16.9)	47.0 (14.3)	.71
Left ventricular end‐diastolic diameter, mm (median [IQR])	56.0 [49.0, 64.0]	55.0 [51.0, 60.5]	57.0 [48.0, 64.0]	.10
Left ventricular end‐systolic diameter, mm (median [IQR])	43.0 [35.0, 49.0]	40.0 [35.0, 48.0]	45.5 [38.8, 53.0]	.18
Left ventricular end‐diastolic volume, ml (median [IQR])	130.0 [101.0, 170.5]	135.0 [100.0, 184.2]	129.5 [101.5, 170.0]	.69
Left ventricular end‐systolic volume, ml (median [IQR])	62.0 [39.0, 92.0]	53.0 [38.7, 89.0]	65.0 [43.8, 98.2]	.40
Mitral regurgitant volume, ml/beat (median [IQR])	46.0 [32.0, 59.0]	49.0 [39.0, 67.0]	42.0 [26.5, 53.8]	.02
Effective regurgitant orifice area, cm^2^ (median [IQR])	0.30 [0.22, 0.43]	0.34 [0.25, 0.54]	0.27 [0.20, 0.38]	.02
Mean mitral valve gradient, mmHg (median [IQR])	2.0 [1.0, 3.0]	2.3 [1.0, 3.0]	2.0 [2.0, 3.0]	.80
Mean RV/RA gradient, mmHg (median [IQR])	35.8 [31.0, 44.2]	36.1 [30.0, 45.1]	35.3 [31.0, 42.5]	.70
TAPSE, mm (mean (SD))	19.1 (4.9)	19.7 (4.8)	18.5 (5.0)	.27
Mitral annular dimension, mm (mean (SD))	35.0 (4.6)	36.7 (4.7)	33.4 (4.0)	<.01
Vena cava inferior diameter, mm (mean (SD))	21.6 (7.6)	20.8 (8.7)	22.5 (6.4)	.39
Tricuspid valve regurgitation grade (%)				
II	20 (23.0)	13 (25.5)	7 (19.4)	.69
III	27 (31.0)	11 (21.6)	16 (44.4)	.04
IV	1 (1.1)	0 (0.0)	1 (2.8)	.86

### 3.1 Procedural outcome

**FIGURE 1:**
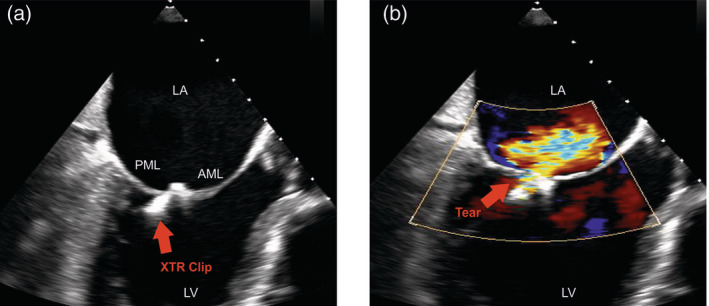
Acute leaflet injury. (A,B) show transesophageal echocardiographic images in one case of acute leaflet tear after implantation of the MitraClip XTR device. The MitraClip XTR device is tilted towards the anterior mitral leaflet (AML) due to tear (B, red arrow) of the posterior mitral leaflet (PML) causing eccentric MR. Left ventricle (LV), left atrium (LA), AML, and PML are labeled accordingly

**FIGURE 2:**
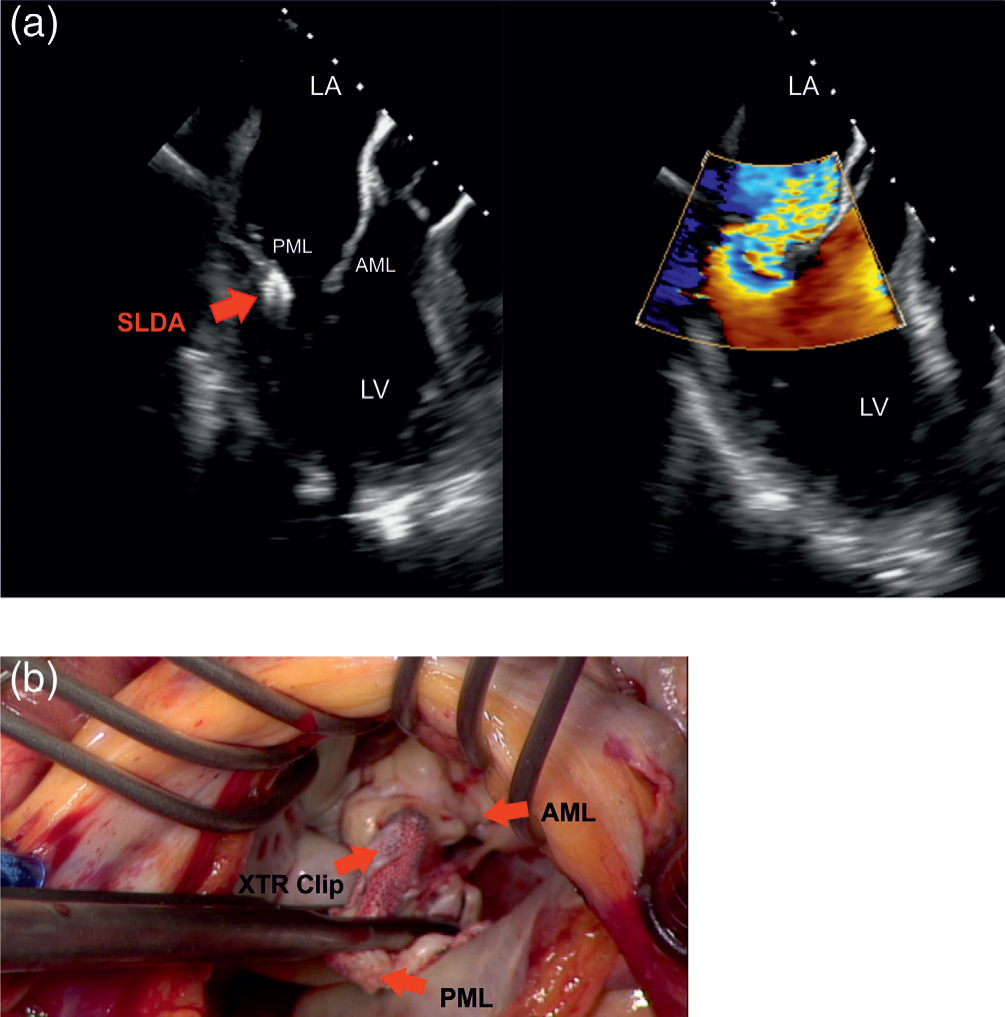
Single leaflet device attachment (SLDA). (A) Shows transesophageal echocardiographic imaging of a patient with SLDA. The MitraClip XTR device (red arrow) is exclusively attached to the posterior mitral leaflet (PML) causing severe MR. (B) Demonstrates an intraoperative image taken during surgery in the same patient. From an atrial perspective one can identify the mitral valve and the MitraClip XTR device held by the surgeon

Acute procedural success (post procedural MR ≤2+) was achieved in 96.1% (49/51 patients) of XTR and in 97.6% (41/42) of NTR treated patients. MR reduction to ≤ I was comparable in both groups (XTR: 70.6% (36/51 patients) vs. NTR: 78.7% (33/42 patients), *p =* .38). The rates for multiple device implantations did not differ between groups (XTR: 58.2% (32/55 patients) vs. NTR: 50.9% (29/57 patients), *p =* .44). Mean MV gradient after successful TMVR did not differ between both groups (XTR: 3.1 mmHg ±1.5, NTR: 3.3 mmHg ±1.5). Considering the composite safety endpoint, there was a significant difference in periprocedural MACCE including leaflet injury (XTR 14.6% vs. NTR 1.7%, 95% CI [2.7, 24.6], *p =* .01, Table S1). In the XTR group, there were 7 patients with leaflet injury, 4 patients with acute leaflet tear and 3 patients with SLDA. Among these patients, 86% (6/7 patients) suffered from a primary valve disease. Additionally, these patients showed increased mitral annular dimensions (37.4 ± 4.8 mm). Echocardiographic imaging of one patient with acute leaflet tear after treatment of degenerative MR is shown in Figure 1(A,B). Following release of the clip, acute tear in the posterior mitral leaflet (PML) was visualized by immediate tilting of the device towards the anterior mitral leaflet (AML) resulting in residual MR (see Videos S1 and S2). Five of these seven patients were successfully treated with additional device implantations. One patient received conservative treatment and one patient with SLDA underwent surgical mitral valve replacement. Intraoperative pictures of SLDA is demonstrated in Figure 2(A,B). One additional XTR treated patient died from a major stroke 5 days after initially successful TMVR.

In the NTR group, there was one case of leaflet injury due to acute leaflet tear. In this case, MR could be reduced to 2+ after implantation of a second clip device. There was no case of SLDA or periprocedural death.

In this study 1/8 XTR patients with periprocedural leaflet tear or SLDA showed calcified mitral annuli prior to TMVR, four patients showed flail leaflets and one patients showed a calcified annulus and a flail leaflet. The only NTR patient with according complications showed a calcified mitral annulus with additional leaflet calcification.

**FIGURE 3:**
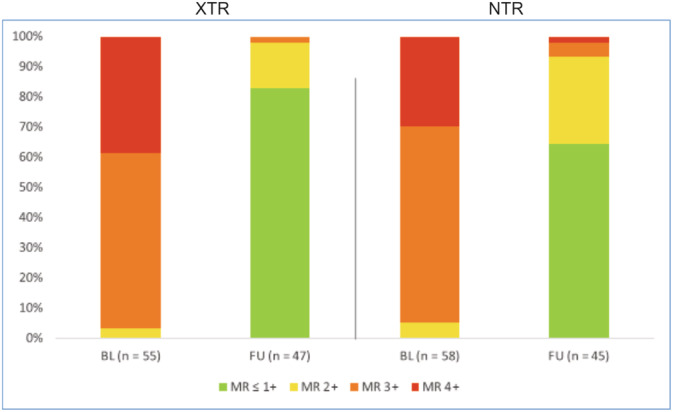
Mitral valve regurgitation grade. Shows the distribution of mitral valve regurgitation grades between baseline and last FU in both groups

Additionally, we assessed mid‐term clinical results after a median follow‐up of 8.5 [4.4, 14.0] months. Data were available in 90.9% (50/55) in the XTR group and in 91.4% (53/58) in the NTR group. MR reduction among these patients remained stable at follow‐up (MR ≤2+ in 91.9% of patients in XTR group and 97% of patients in NTR group, *p =* .31, Figure 3). Accordingly, patients showed persistent clinical improvement. At the time of last follow‐up, the rate of patients with NYHA functional class ≥III has decreased significantly from 89.1% at baseline to 20.6% at follow‐up (*p <* .001) in patients treated with MitraClip XTR. This was accompanied by a significant increase in 6‐min walk test (282.9 m ± 151 vs. 413 m ± 324, *p =* .04) and a notable decrease in NTproBNP levels (3015 pg/ml [1271, 7587] vs. 2146 pg/ml [757, 4464], *p =* .07). Patients in the NTR group showed similar clinical results. The rate of patients with NYHA functional class ≥III decreased significantly from 96.6% at baseline to 15.6% at follow‐up (*p <* .001) while physical capacity evaluated by 6‐min walking distance showed significant improvement (216 m ± 129 vs. 250 m ± 120, *p =* .03). NTproBNP levels did not differ between baseline and follow‐up among NTR patients (2656 pg/ml [1436, 4960] vs. 2439 pg/ml [1464, 6303]). Additional echocardiographic follow‐up assessment revealed a durable improvement of quantitative parameters of MR severity in both groups. There was a notable decrease in mitral annular diameter (MAD) (37.0 mm ±4.7 vs. 33.0 mm ±3.6, *p <* .001) and mean RV/RA gradient (41.0 mmHg ±15 vs. 34.0 mmHg ±15, *p =* .04) at follow‐up in both groups (Tables S2, S3) an. Echocardiographic outcome measurements for both groups are shown in Tables S2 and S3. The additional secondary endpoint mid‐term term survival showed no significant difference between both treatment groups (HR for death 2.14 (95% CI, 0.65–3.07, *p =* .39, Figure S1).

## 4 DISCUSSION

This study systematically analyses and compares the efficacy and safety of two MitraClip‐devices, the MitraClip XTR and NTR system. While both devices effectively reduced MR severity and associated symptoms, more leaflet injuries (leaflet tears and SLDA) were observed in the XTR group. After gaining approval by the Food and Drug Administration in 2013, TMVR with the MitraClip system has been established as an alternative treatment approach especially for selected patients with elevated perioperative risk.^9^, ^13^, ^21^, ^22^ Compared with the initially restrictive inclusion criteria of the EVEREST II trial, now far more patients with rather complex anatomies are eligible for this procedure.^23^ With the introduction of the new MitraClip XTR system in 2018, even larger coaptation gaps and larger flails can be effectively treated.^13^ Although both MitraClip devices demonstrated effective reduction of MR, some authors reported incidents of leaflet injury using the MitraClip XTR device, which might result from increased tension exerted on the valve leaflets.^13^, ^24^ Therefore, the objective of the present study was to compare the efficacy and safety of the two MitraClip systems (XTR vs. NTR) in a real world setting.

In this study, both groups showed significant and persistent reduction of MR at follow‐up assessed by echocardiographic imaging. Acute procedural success in this study (96.7% MR ≤ II, 74.2% MR ≤ I) was similar to the results reported in MITRA‐FR (76% MR ≤ I) and COAPT trial (95% ≤ II). Despite being higher than reported by registries and retrospective studies (37–43%),^12^, ^13^ the proportion of patients with multiple device implantations in this study (55%) appears to be more comparable to the MITRA‐FR (54%) and COAPT trial (62%). Interestingly, the usage of the XTR device does not seem to reduce the number of devices needed per patient.

Both, XTR and NTR system managed to provide mid‐term reduction of heart‐failure related symptoms assessed by NYHA functional class and additional echocardiographic imaging confirmed durable MR reduction. Previous in vitro and in vivo assessments of the MitraClip system and especially a recent study on the XTR system reported few incidents of leaflet injuries requiring surgical intervention.^13^, ^25^, ^26^ These few events often led to SLDA.^14^, ^15^ Praz et al. reported leaflet damage to be accidental while hypothesizing it might result from increased tension on the leaflets exerted by longer clip arms of the XTR system. While leaflet injuries generally seem to be rare events, we can identify higher rates of device complications after MitraClip XTR implantation in our study cohort. These patients especially suffered from degenerative valve disease with increased mitral annular dimensions. Leaflet injuries in this series occurred despite being aware of increased tension on the mitral leaflets. Clips were closed slowly and the system was moved towards the left ventricle during the grasping process in order to minimize tension on the leaflets. In this study a high proportion of patients with periprocedural leaflet injury had calcified annuli or flail leaflets. In this context, in particular patients with fragile leaflets and/or calcified mitral annuli might be preferentially treated using the MitraClip NTR system. Furthermore, our data indicate that the MitraClip NTR system should be preferred even in patients with degenerative valve disease whenever possible. However, in patients with primary MR with broad flail leaflets or secondary MR with extreme annular dilatation and large coaptation gaps, the MitraClip XTR System might be selected if its implantation is handled with caution. Our findings underline the importance of accurate periprocedural 2‐ and 3‐dimentional transthoracic and transesophageal echocardiography to assure selection of ideal candidates.

Limitations are the retrospective nature of this study leading to imbalanced baseline characteristics, low patient number as well as the incomplete follow‐up. Furthermore, there was no independent echo core lab analysis. However, to the best of our knowledge, this is the first study comparing different device sizes for edge‐to‐edge mitral valve therapy. Further multicenter studies are on the way to define the optimal device selection for TMVR. In this context the recently introduced PASCAL system (Edwards Lifesciences, Irvine, CA) as well as the next generation of MitraClips, that have the capability to perform independent grasping, need to be taken in consideration. This study highlights the necessity of a careful and individual patient selection by an interdisciplinary heart team to assure an optimal treatment approach.

## 5 CONCLUSION

TMVR with both, MitraClip XTR and MitraClip NTR, appears to be effective and durable. Moreover, TMVR reduces HF related symptoms and improves functional capacity. In a real‐world setting, the MitraClip XTR system appears to have higher rates of device‐associated complications than the NTR system. The increased amount of tissue grasping by the XTR system may increase the risk of acute leaflet injury. Therefore, the MitraClip XTR system should primarily be used in selected patients with complex anatomies.

## CONFLICT OF INTEREST

Daniel Braun, Mathias Orban and Michael Nabauer received speaker honoraria from the Abbott Vascular. Joerg Hausleiter received speaker honoraria from and serves as consultant for Abbott Vascular and Edwards Lifesciences.

## Supporting information


**Figure S1** Mid‐term survival: This Kaplan–Meier graph displays the mid‐term survival in both groups assessed by telephone calls and local residents' registration office.

**Table S1**: Supplementary information.

**Video S1**: Intraprocedural transesophageal echocardiographic imaging showing tilting of the MitraClip XTR device towards the anterior mitral leaflet after clip release due to PML tear.

**Video S2**: Transesophageal echocardiographic imaging demonstrating the leaflet tear associated residual MR after implantation of a second MitraClip XTR device.

## Data Availability

Data available on request from the authors.
