# Supplementary figures and images for: Percutaneous edge‐to‐edge repair of severe mitral regurgitation using the MitraClip XTR versus NTR system
Source: Clin Cardiol. 2021 Mar 24;44(5):708–14. doi: 10.1002/clc.23599 (PMC8119798; doi:10.1002/clc.23599)

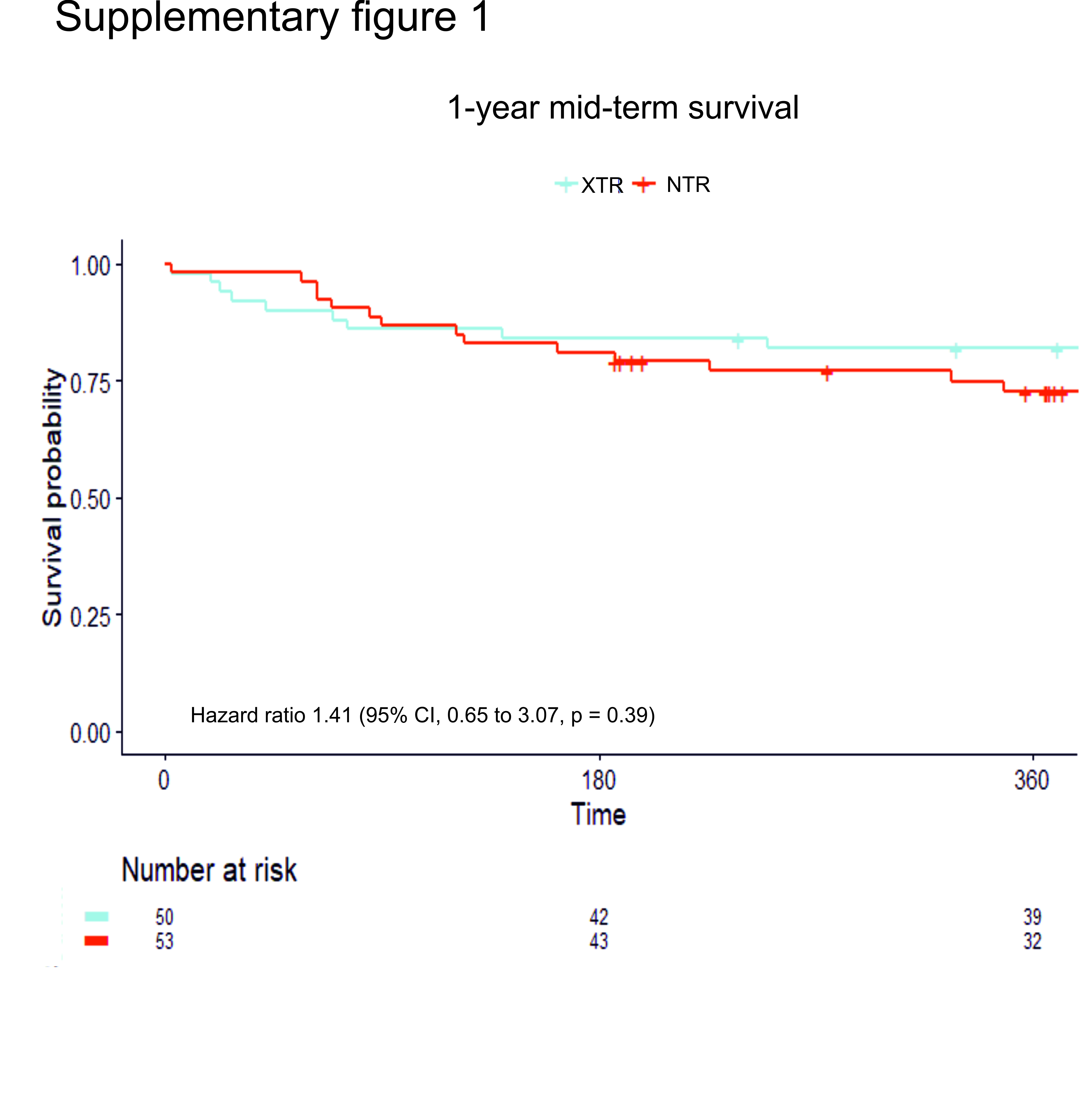

Supplement: Supplementary file 1 — Figure S1 Mid‐term survival: This Kaplan–Meier graph displays the mid‐term survival in both groups assessed by telephone calls and local residents' registration office. [file CLC-44-708-s002.tif]
