# Supplementary material for: Percutaneous edge‐to‐edge repair of severe mitral regurgitation using the MitraClip XTR versus NTR system
Source: Clin Cardiol. 2021 Mar 24;44(5):708–14. doi: 10.1002/clc.23599 (PMC8119798; doi:10.1002/clc.23599)
Supplement: Supplementary file 2 — Table S1: Supplementary information. [file CLC-44-708-s001.docx]

**Supplementary tables**
